# Tracking the spatial diffusion of influenza and norovirus using telehealth data: A spatiotemporal analysis of syndromic data

**DOI:** 10.1186/1741-7015-6-16

**Published:** 2008-06-26

**Authors:** Duncan L Cooper, Gillian E Smith, Martyn Regan, Shirley Large, Peter P Groenewegen

**Affiliations:** 1 Bradford and Airedale tPCT, West Yorkshire, UK; 2 Health Protection Agency West Midlands, Birmingham, UK; 3 Health Protection Agency East Midlands, Nottingham, UK; 4 NHS Direct, Hampshire, UK; 5 Netherlands Institute for Health Services Research, Utrecht, Netherlands

## Abstract

**Background:**

Telehealth systems have a large potential for informing public health authorities in an early stage of outbreaks of communicable disease. Influenza and norovirus are common viruses that cause significant respiratory and gastrointestinal disease worldwide. Data about these viruses are not routinely mapped for surveillance purposes in the UK, so the spatial diffusion of national outbreaks and epidemics is not known as such incidents occur. We aim to describe the geographical origin and diffusion of rises in fever and vomiting calls to a national telehealth system, and consider the usefulness of these findings for influenza and norovirus surveillance.

**Methods:**

Data about fever calls (5- to 14-year-old age group) and vomiting calls (≥ 5-year-old age group) in school-age children, proxies for influenza and norovirus, respectively, were extracted from the NHS Direct national telehealth database for the period June 2005 to May 2006. The SaTScan space-time permutation model was used to retrospectively detect statistically significant clusters of calls on a week-by-week basis. These syndromic results were validated against existing laboratory and clinical surveillance data.

**Results:**

We identified two distinct periods of elevated fever calls. The first originated in the North-West of England during November 2005 and spread in a south-east direction, the second began in Central England during January 2006 and moved southwards. The timing, geographical location, and age structure of these rises in fever calls were similar to a national influenza B outbreak that occurred during winter 2005–2006. We also identified significantly elevated levels of vomiting calls in South-East England during winter 2005–2006.

**Conclusion:**

Spatiotemporal analyses of telehealth data, specifically fever calls, provided a timely and unique description of the evolution of a national influenza outbreak. In a similar way the tool may be useful for tracking norovirus, although the lack of consistent comparison data makes this more difficult to assess. In interpreting these results, care must be taken to consider other infectious and non-infectious causes of fever and vomiting. The scan statistic should be considered for spatial analyses of telehealth data elsewhere and will be used to initiate prospective geographical surveillance of influenza in England.

## Background

Disease surveillance systems are used for identifying important events and trends within the distribution of known diseases. This information should inform and drive public health action. Traditionally, laboratory reports and clinical diagnoses are used for disease surveillance, providing epidemiological details about the time, person and place associated with disease. In recent years there has been a growth in syndromic surveillance systems that collect and analyse pre-diagnostic data to provide an early warning of infectious disease outbreaks or potential bio-terrorist attacks [1]. These systems have been successful in detecting rises in syndromes associated with common viral diseases [2,3]. Influenza and norovirus are two such viruses that cause significant respiratory and gastrointestinal disease in the UK [4,5] and worldwide. Both are responsible for outbreaks within healthcare [6] and educational settings [7], and are associated with significant economic costs [8,9].

The influenza virus causes an acute infection of the respiratory tract characterised by cough, fever and myalgia, and in more serious cases can lead to pneumonia. Its epidemiology has been studied in great detail, partly due to the ability of the virus to mutate, causing national epidemics and worldwide pandemics (for example, during 1918, 1957 and 1968). On a population level the disease spreads via a combination of contagious diffusion (wave-like from a central or multiple foci) and hierarchical diffusion (movement from large to smaller towns) [10]. During the 1918–1919 pandemic in the UK influenza moved in a southerly, then northerly, then southerly direction during three successive epidemic waves [11]. Although there has been a secular decline in the incidence of influenza-like illness (ILI) in the UK in recent decades [4], elevated activity still occurs during winter, often reaching peak levels first in Northern England [7,12]. For example, the winter of 2005–2006 was marked by a national outbreak of influenza B and nearly 700 reported school outbreaks during January and February. A well-established surveillance programme [13] published weekly reports throughout the winter, summarising clinical incidence and laboratory data and tabulating regional levels.

Norovirus is associated with projectile vomiting and diarrhoea. There are an estimated 700,000 cases in England each year [5], although it is estimated that only one out of every 1600 cases reaches national laboratory surveillance [14], prompting calls for methods of supplementing current surveillance systems. Like influenza, new strains of norovirus may emerge [15], causing significant illness and a rise in reported outbreaks. The geographical variation in community infection rates of norovirus is poorly understood in the UK as the 'gold standard' for surveillance is considered to be outbreak reports. These reports originate mainly from institutional rather than community settings [6] and are neither timely nor reported in a consistent manner across the country.

Neither influenza nor norovirus data are routinely mapped for surveillance purposes in the UK. This means that the local incidence, and the spatial diffusion of national epidemics, cannot be described prospectively for public health teams, health service planners and the public. The Health Protection Agency (HPA) currently collects and analyses data from a national telephone triage service (NHS Direct) for syndromic surveillance purposes [16]. NHS Direct covers the whole of England and Wales and is widely used by the population. NHS Direct nurses record demographic and syndromic call details over the telephone so the call data offer potential for disease surveillance. Therefore, analysing the usefulness of NHS Direct data has a wider relevance for public health and preparedness.

In this article we have used syndromic data about NHS Direct fever and vomiting calls, of which there are many infectious and non-infectious causes. On a national and regional level, NHS Direct fever calls for school-age children rise at the same time as laboratory reports of influenza B [17]. Vomiting calls rise during periods of increased norovirus activity nationally [18]. Developments in NHS Direct data management systems now enable syndromic call data to be extracted and analysed to a local level. In this study we aimed to characterise the geographical origin and diffusion of rises in NHS Direct fever and vomiting calls (that is, potential epidemics). Freely available spatiotemporal analyses software (SaTScan) was used to detect clusters of elevated NHS Direct calls on a week-by-week basis. We compared these syndromic results with existing laboratory and clinical surveillance data in order to determine whether NHS Direct data provide a sensitive and timely means of describing local and national level increases in common viruses.

## Methods

### Data collection

Data were extracted from the NHS Direct national database about the syndrome, time of call, age group, and postcode district (geographical identifier) of each NHS Direct call in England for the period June 2005 to May 2006. At the time of analysis a single year's postcoded data were available. A geographical information system (MapInfo GIS) was used to assign a Primary Care Trust (PCT) code to each call. The PCT is the local unit of administration for primary and public health services in England. There are 151 PCTs with an average population of 353,000 people. Two subsets of NHS Direct data were extracted: fever calls for the 5- to 14-year-old age group, used as a proxy for influenza B infection, which predominantly affects school-age children; and vomiting calls for the ≥ 5-year-old age group, used as a proxy for norovirus infection. Vomiting calls about those younger than 5 years old were excluded from the analysis to remove the potentially confounding effect of rotavirus (another common viral cause of vomiting which almost exclusively affects children under 5 years old). It is not currently possible to extract multiple symptoms linked to individual callers due to the way in which call details are stored within the NHS Direct call database.

### Statistical methods

Data were analysed using the SaTScan scan statistic to analyse spatial, temporal or spatiotemporal data [19]. The scan statistic uses circular windows (potential cluster areas) starting as a single point (PCT location) and increasing in radius to form an additional circle each time a new point is reached. The maximum spatial extent and time period of the circles is defined by the user; in this case it was set to extend to 50% of the total population of England. For each circle the number of observed and expected cases is calculated as well as a likelihood ratio and relative risk (RR) to determine whether the call rate inside the circle is greater than the area outside (that is, a cluster). The statistical significance of clusters with an excess of cases (observed > expected) is calculated by using Monte Carlo hypothesis testing to compare the real data against randomly generated datasets. Clusters with a significantly elevated RR (*p *≤ 0.05) were stratified into five levels of risk and mapped using a GIS. If a PCT was contained within two overlapping clusters the RR with the lowest *p*-value was mapped, a method borrowed from Boscoe et al to visualise SaTScan output [20]. The application of the scan statistic to communicable disease surveillance has been described extensively elsewhere [21-24].

### Analyses

We used a three-stage process to address our aims. For stage 1 we mapped annual total NHS Direct call rates by PCT and used the SaTSCan scan statistic to identify areas of high call rates all year round ('global clustering'). Global clustering may bias the results of the weekly analyses (stage 2) by causing frequent but misleading clusters of syndromic calls in areas of high total call rates. Accounting for the results of stage 1, stage 2 was a spatiotemporal analysis of syndromic calls to describe the location and diffusion of significant rises in fever and vomiting calls. Stage 3 used existing influenza and norovirus surveillance sources (GP consultations, laboratory reports, outbreaks) to describe the accuracy and usefulness of NHS Direct syndromic data. A similar staged approach has been used previously to identify clusters of *Escherichia coli *0157 cases in Canada [24].

### Stage 1: mapping total NHS Direct call rates

Total annual NHS Direct call rates (all ages, 5 to 14 years and ≥ 5 years) were mapped by PCT. We then conducted a single retrospective spatial analysis of one year's call data using the SaTScan Poisson probability model. This tested for areas with significantly high or low annual call rates over the whole study period. Three data files were required for analysis: the number of calls per week in each PCT (numerator), the population of each PCT (denominator), and the grid coordinates of the centroid of each PCT (location). The PCT was preferred as the unit of analysis (rather than the smaller postcode district) to test provision of PCT-based data for seasonal influenza surveillance and pandemic influenza preparedness, and to maintain statistical stability by avoiding generating clusters with very small numbers of calls, which in practice are unlikely to be investigated further. This choice of geographical unit is discussed later in the article.

### Stage 2: spatiotemporal analyses of syndromic calls

The results of stage 1 (described in the results section) indicated that there was significant global clustering and spatial heterogeneity in local call rates. To correct for this underlying spatial variation, the SaTScan space-time permutation model was used to conduct further analyses. This approach requires only case data (in this case either fever or vomiting calls) and adjusts for purely spatial or purely temporal clusters. For this method the number of observed calls in a cluster is compared with the expected number, assuming that the spatial and temporal locations of calls are independent of each other (no space-time interaction). At each time interval specified, a cluster is identified if a specific area has a higher proportion of excess cases than surrounding areas. Therefore areas with historically high usage of NHS Direct (identified in stage 1) will not bias the results. Also, if during a specific week (for example, the Christmas holiday when doctors' surgeries are closed) all areas experience a doubling of vomiting calls, no clusters will be identified. Such a national rise would be detected by the existing NHS Direct syndromic surveillance system [16]. Changes in PCT population over the study period could hypothetically cause clusters in areas with increased population and reduce the probability of clusters in areas with decreased population. Our study period covered a single year so population change is considered negligible.

We used the space-time permutation model (on historical data) to mimic prospective surveillance and identify 'live' clusters (clusters ending with the most recent date) lasting 1 week and with a *p*-value of *p *≤ 0.05. We retained the maximum cluster population size of 50%. SaTScan uses cases (calls) as a proxy for population in the space-time permutation model as only case data are included. The first 4 months' data (1 June 2005 to 2 October 2005) were used as the control period on which the scan statistic calculates an expected count for each location. Repeated analyses were performed for each additional week's data for a test period from 3 October 2005 to 21 May 2006 for fever calls (5 to 14 years) and vomiting calls (≥ 5 years) separately. Although daily NHS Direct data were available we conducted weekly analyses to simplify the presentation of data and align temporal comparisons with clinical and laboratory data which are available weekly. Details of the location, observed and expected calls, and level of significance of each significant space-time cluster were recorded. The ratio of observed to expected calls (O/E) for each cluster was mapped for each weekly analysis using the same method as in stage 1.

### Stage 3: comparison against existing surveillance data

The dates and spatial distribution of fever clusters (identified in stage 2) were compared against: routinely available influenza surveillance data; weekly ILI rates for the 5- to 14-year-old age group for Northern, Central and Southern England (obtained from the Royal College of General Practitioners Weekly Returns Service) [25]; and the weekly number (all ages combined, influenza A and B separately) of positive influenza samples from community sources from the HPA Centre for Infections virological surveillance scheme [13]. The dates and spatial distribution of vomiting clusters were compared against weekly numbers of norovirus outbreaks recorded by the HPA Centre of Infections Modular Open Laboratory System (MOLIS), presented by the region of origin of laboratory samples. The MOLIS database was recommended as a suitable source of outbreak data as each laboratory sample has an outbreak code and report date, making it possible to derive the number of outbreaks per week and region.

## Results

During the study period there were 4,728,939 NHS Direct calls from the public seeking advice for all symptoms of which 4,406,607 (93%) were linked via a postcode to a PCT. The total call rate was 82.1 calls per 1000 people, ranging from 33.9 to 212 per 100,000 between PCTs. Annual syndromic calls for fever (5 to 14 years) and vomiting (≥ 5 years) are summarised in Table 1.

**Table 1:** Numbers, rates per 1000/year and percentages of syndromic calls (June 2005 to May 2006)

	Number of calls	Call rate per 1000/year (range within Primary Care Trusts)	Syndromic calls, as a percentage of total calls for same age group
Vomiting calls (≥ 5 years)	88,452	1.8 (0.6 to 3.2)	2.5%
Fever calls (5 to 14 years)	23,431	3.7 (1.1 to 8.5)	6.4%

### Stage 1

**Figure 1:**
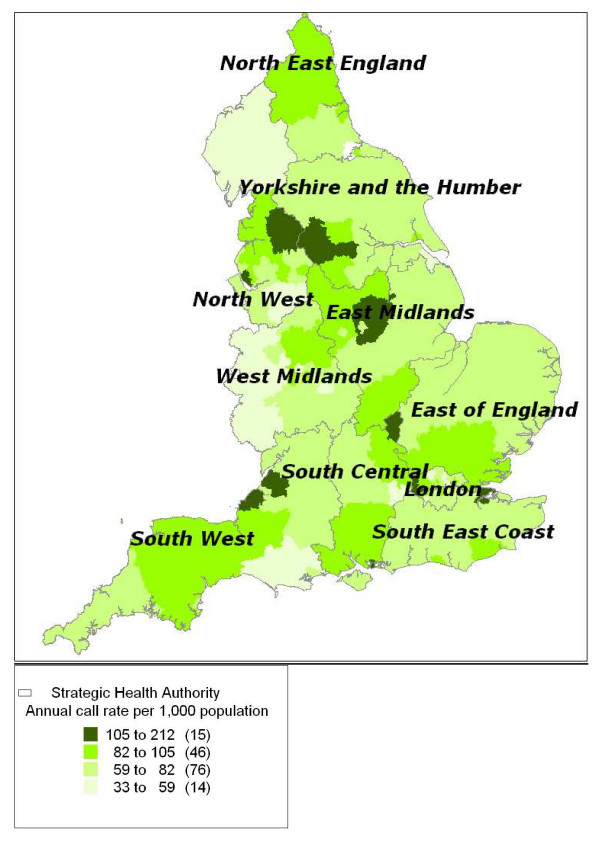
**Annual total call rate per 1000 population**. Annual total call rate per 1000 population per year (June 2005 to May 2006) mapped by Primary Care Trust.

**Figure 2:**
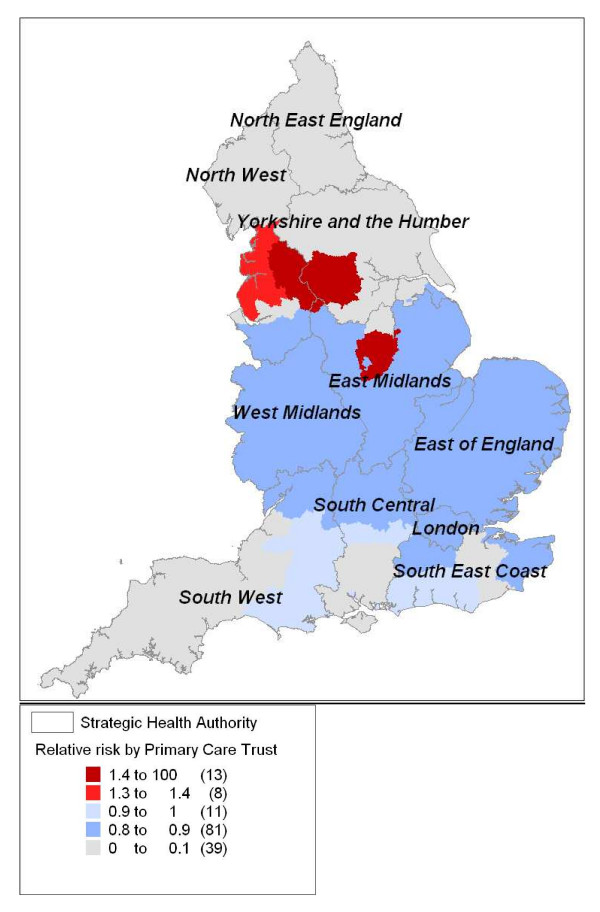
**Areas of significantly high or low annual total call rates**. Areas of significantly high or low annual total call rates (June 2005 to May 2006) displayed as relative risks and mapped by Primary Care Trust.

The highest all-symptom annual call rates (in excess of 100 calls per 1000/year) were found particularly in the North-West and Yorkshire and Humber regions, with other areas of high incidence areas scattered throughout England (Figure 1). Spatial analyses of these data highlighted two areas of significantly high call rates (RR: 1.3 to 1.5; *p *= 0.001) in Northern England and Nottinghamshire, and a widespread area of significantly low call rates across Central and Eastern England (RR: 0.8 to 0.9; *p *= 0.001); see Figure 2. Total call rates for the 5- to 14-year-old and ≥ 5-year-old age groups exhibited a similar geographical variation by PCT (not presented).

The significant geographical variation in total call rates meant that if we had adopted a similar SaTScan model for the iterative weekly analyses of syndromic data (stage 2) the results would have been largely determined by this underlying variation in call rates. The space-time permutation model was used, therefore, whereby a baseline period is used to establish the expected weekly number of syndromic calls in each PCT.

### Stage 2

**Figure 3:**
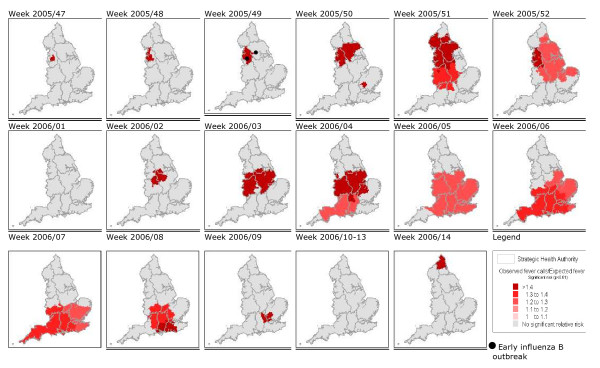
**Areas with significantly high numbers of fever calls**. Areas with significantly high numbers of fever calls (clusters) displayed as observed/expected ratios by week and location of the first reported influenza B outbreaks. There were no significant clusters prior to week 47 in 2005 and after week 14 in 2006 during our test period.

There were 27 fever clusters during the study period (*p *≤ 0.01), all but one within a 15-week period between November 2005 and March 2006 (Table 2). The clusters ranged from 2 to 72 PCTs in size (mean 34), and from 18 to 937 fever calls (mean 310). The shaded areas on the maps (Figure 3) denote areas with statistically high observed numbers of fever calls ('clusters') compared with the expected number. The first significant cluster was detected in North-West England during week 47 in 2005. This area of raised fever incidence increased in size to cover large areas of Central and Northern England by week 52 in 2005. A second rise in fever calls began with a cluster in Central England during week 02 in 2006 and spread south, reaching its maximum extent during week 07 in 2006. Isolated clusters lasting a single week were detected in Hertfordshire (week 50 in 2005) and Northumberland (week 14 in 2006).

**Table 2:** Description of all significant fever clusters (*p *≤ 0.05) detected by the scan statistic during our test period (23 October 2005 to 21 May 2006)

Year	Week	Location	Number of PCTs involved	Observed calls (O)	Expected calls (E)	O/E	*p*-value
		(centroid PCT)	(radius in km)		[based on control period]		
2005	47	Ashton, Leigh and Wigan	8 (20)	34	15	2.23	0.005
	48	Warrington	8 (22)	42	20	2.15	0.002
	49	North Lancashire	11 (61)	71	31	2.27	0.001
	49	Salford	19 (39)	100	57	1.75	0.001
	50	East Lancashire	29 (72)	214	125	1.71	0.001
	50	East and North Hertfordshire	2 (22)	18	5	3.33	0.001
	51	North Lancashire	50 (147)	394	245	1.6	0.001
	51	South Staffordshire	59 (125)	404	309	1.31	0.01
	52	Wirral	22 (71)	156	100	1.56	0.001
	52	Hull	72 (185)	347	278	1.25	0.008
2006	02	Derbyshire County	12 (50)	114	66	1.74	0.001
	03	Nottingham City	21 (75)	266	170	1.56	0.001
	03	Telford and Wrekin	20 (74)	191	135	1.42	0.001
	04	Leicester City	31 (92)	405	276	1.47	0.001
	04	Shropshire County	15 (64)	175	110	1.59	0.001
	04	Bath and North East Somerset	31 (140)	325	251	1.29	0.002
	05	Gloucestershire	76 (154)	937	722	1.30	0.001
	05	Mid Essex	72 (185)	879	720	1.22	0.001
	06	Southampton city	69 (164)	889	661	1.34	0.001
	06	North East Essex	66 (187)	855	660	1.30	0.001
	07	Torbay	32 (250)	337	251	1.34	0.001
	07	East Sussex Downs and Weald	68 (194)	549	453	1.21	0.003
	08	West Sussex	18 (64)	117	72	1.61	0.001
	08	Oxfordshire	38 (87)	201	149	1.35	0.01
	09	West Hertfordshire	21 (34)	124	74	1.68	0.001
	09	Berkshire West	30 (76)	212	148	1.43	0.001
	14	Northumberland	6 (62)	33	13	2.46	0.001

PCT, Primary Care Trust.

**Figure 4:**
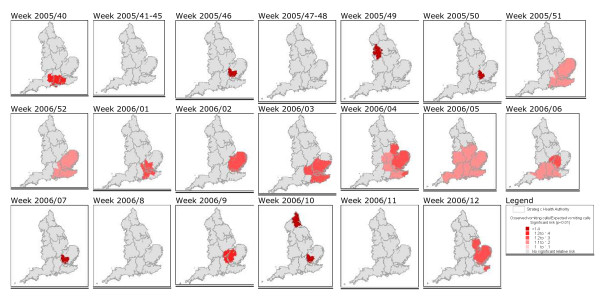
**Areas with significantly high numbers of vomiting calls**. Areas with significantly high numbers of vomiting calls (clusters) displayed as observed/expected ratios by week. There were no significant clusters prior to week 40 in 2005 and after week 12 in 2006 during our test period.

There were 22 vomiting clusters (*p *≤ 0.01), all but three within a 14-week period between December 2005 and March 2006 (Table 3). The clusters ranged from 4 to 74 PCTs in size (mean 32), and from 85 to 1345 vomiting calls (mean 555). Sporadic 1-week clusters of vomiting calls were observed in the North-West and Southern England between weeks 40 and 50 in 2005. A widespread significant rise in vomiting calls occurred in South-East England in week 51 of 2005, reaching its maximum extent in week 05 of 2006 (Figure 4).

**Table 3:** Description of all significant vomiting clusters (*p *≤ 0.05) detected by the scan statistic during our test period (23 October 2005 to 21 May 2006)

Year	Week	Location	Number of PCTs involved	Observed calls (O)	Expected calls (E)	O/E	*p*-value
		(centroid PCT)	(radius in km)		[based on control period]		
2005	40	Portsmouth City Teaching	11 (81)	229	171	1.34	0.006
	46	East and North Hertfordshire	9 (33)	121	80	1.52	0.002
	49	Suffolk	31 (113)	367	292	1.26	0.004
	50	Luton	4 (22)	85	47	1.79	0.001
	51	Bedfordshire	5 (37)	147	86	1.7	0.001
	51	Hounslow	41 (59)	811	687	1.19	0.001
	52	Eastern and Coastal Kent	59 (167)	1354	1204	1.12	0.007
2006	01	Hammersmith and Fulham	43 (64)	744	612	1.22	0.001
	02	Suffolk	36 (115)	525	412	1.27	0.001
	03	Bexley	56 (113)	896	737	1.21	0.001
	04	Great Yarmouth and Waveney	29 (165)	429	331	1.29	0.001
	04	Milton Keynes	55 (93)	799	688	1.16	0.009
	05	South Gloucestershire	65 (161)	1023	860	1.19	0.001
	05	Brighton and Hove City	46 (97)	789	662	1.19	0.001
	05	Cambridgeshire	74 (144)	1177	1046	1.12	0.009
	06	Southampton city	44 (116)	822	689	1.19	0.002
	06	Bedfordshire	26 (67)	480	394	1.22	0.007
	07	East and North Hertfordshire	18 (41)	293	214	1.37	0.001
	09	Bedfordshire	11 (52)	249	183	1.36	0.002
	10	East and north Hertfordshire	12 (37)	241	164	1.47	0.001
	10	Newcastle	8 (46)	156	108	1.45	0.002
	12	Great Yarmouth and Waveney	31 (167)	469	370	1.27	0.001

PCT, Primary Care Trust

### Stage 3

**Figure 5:**
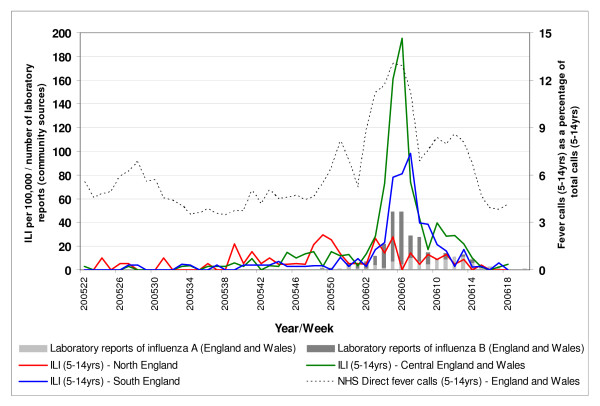
**Weekly GP influenza-like-illness consultation rates**. Weekly GP influenza-like-illness consultation rates for the 5- to 14-year-old age group for Northern, Central and Southern England; weekly numbers (all ages combined) of positive influenza samples (influenza A and B separately) from community sources; and NHS Direct fever calls as a percentage of total calls (5 to 14 years) for England and Wales.

The pre-Christmas significant cluster of NHS Direct fever calls (5 to 14 years) in North-West England (weeks 47 and 48 of 2005) occurred prior to a pre-Christmas rise in GP-diagnosed ILI (5 to 14 years) in Northern England (peaking in week 50 of 2005) and reports of early influenza B outbreaks in primary schools in the North-West reported in week 49 of 2005 (Figure 5) (see [7]). The second significant rise in NHS Direct fever calls originated in Central England during week 02 of 2006, 2 weeks prior to a rise in ILI above baseline levels in Central England (30 per 100,000, see [4]). The subsequent southerly movement and expansion of this rise in fever calls during weeks 03 to 07 in 2006 occurred concurrently with a rise in ILI peaking first in Central England (week 06 in 2006) and then Southern England (week 07 in 2006), and a rise in laboratory reports of influenza B peaking in weeks 05 and 06 of 2006. A rise in laboratory reports is defined as five or more laboratory reports during two consecutive weeks [4]).

The majority of the norovirus outbreaks from the MOLIS database were from the North-West, South-East and London regions (84%). There was a period of elevated numbers of outbreaks in the London and South-East regions between November-December 2005 and March 2006 (week 02 in 2006 peak was 26 outbreaks) and a later peak in North-West outbreaks during April (week 15 in 2006 peak was 34 outbreaks). The period of significantly high vomiting calls in South-East England was contained within this period (December 2005 to mid-February 2006) with localised vomiting clusters in South-East England lasting until March 2006.

## Discussion

Spatiotemporal analyses of telehealth calls may serve as a useful adjunct to influenza surveillance. Our analyses of NHS Direct fever calls have provided a clear and detailed geographical description of seasonal influenza in England in 2005–2006. We have identified two distinct periods of significantly high NHS Direct fever calls regarding school-age children. The first rise originated in North-West England during late November 2005 and spread in a predominantly eastwards and then southwards direction over the following month. The second rise began in Central England during mid-January 2006 and moved southwards to eventually cover all of southern England. The timing and geographical location of these rises in fever calls appear similar to a national influenza B outbreak occurring during winter 2005–2006 affecting the same age group, although they were visible slightly earlier than in other data sources. We also identified significantly elevated numbers of vomiting calls in Central and South-East England during December 2005 to February 2006, within a longer period of elevated norovirus outbreak reports in the South-East. There was no apparent spatial correlation between vomiting calls and an April 2006 rise in norovirus outbreaks in the North-East of England. Assessment of the usefulness of vomiting call data for monitoring the spread of norovirus was limited by inconsistent regional level outbreak sample data within our norovirus data source.

We discuss these results from two perspectives. The first and general perspective relates to the potential of a telephone triage system, such as NHS Direct, for surveillance purposes; the second perspective for discussion concerns the specific findings and limitations of the current spatial analysis.

### The potential of telephone triage systems for surveillance

Telephone triage health systems may be useful for surveillance purposes for a number of reasons. These services are generally available to the entire population of the area covered and, in the case of NHS Direct, are centrally managed. This means that clinical algorithms are chosen in a consistent manner between call centres (although experienced NHS Direct nurses may be more likely to provide self-care advice than more junior colleagues [26]). Second, the data are available on a daily basis as opposed to GP- and laboratory-based surveillance systems, which generally report weekly. Third, telehealth is many people's first or only point of contact with the health service, providing an early opportunity to identify an increase in illness potentially 'under the radar' of other systems.

Despite the above, call data will only provide meaningful intelligence if callers' reported illness is representative of community morbidity. In England and Wales, total NHS Direct call rates (82 per 1000 in England) are approximately 3% of annual GP consultation rates for all illness (2700 consultations per 1000; A. Elliot, personal communication). One-quarter of the population have used NHS Direct [27]. Our work identified areas of significantly high NHS Direct call rates in Northern England and substantial variation between PCTs (ranging from 33 to 212 calls per 1000 people per year). This disparity is influenced by a combination of factors, including local arrangements for provision of GP out-of-hours services (generating on average 160 calls per 1000 people per annum [28]), social deprivation [29] and the length of time the local NHS Direct site has been in operation [27].

NHS Direct receives a disproportionately high number of calls about children under 4 years old (four times higher than the total call rate) and women of child-bearing age (twice the male call rate). Both of these groups traditionally have a high health service utilisation in the UK [30]. This reporting tendency could have advantages for timely sentinel surveillance of children, to provide early warning of more widespread population impacts. Older people, who have high GP and hospitalisation rates, use NHS Direct the least of any age group. This age and gender bias is consistent with large public telephone triage systems in New Zealand [31], Australia [32] and Canada [33]. NHS Direct data are, therefore, most suited to surveillance of common illnesses in the UK for those aged below 65 years. Measures must be taken to account for spatial variation in usage, such as establishing local baselines from which to detect real variation in call incidence (as employed in this study using a scan statistic).

Prospectively NHS Direct telephone triage data have been used to identify sharp rises in syndromes at a regional and national level [16,17]. The retrospective analysis reported here has demonstrated that it is also possible to identify a sub-regional rise in calls suggestive of influenza infection. Other potential benefits include the use of the data to inform the NHS Direct responsive messaging service, an operational tool designed to help manage service demand and to inform patients of relevant topical information (for example, 'flu and colds', 'diarrhoea and vomiting'). Trends in calls may either act as a trigger to initiate responsive messages or provide statistical support for the ongoing provision of this health advice. In addition, as well as monitoring calls suggestive of infections, these data are also used for the acute response to major incidents. For example, during July 2007, daily surveillance of calls identified a statistically significant 40% rise in calls in the Gloucester flood region (caused in part by a rise in people seeking health information and advice). This information was used to brief a national incident team and the Government on the health effects of the floods.

A qualitative evaluation has explored the usefulness of these telephone triage data for surveillance amongst a sample (*n *= 91) of users of the system (those that receive surveillance bulletins and 'alerts') [34]. The most commonly cited benefits of the surveillance were: providing real-time information for incident teams, local PCTs, media messages, and question and answer briefings for GPs and the public; supporting national plans (for example, surveillance of heat stroke calls during the heatwaves of 2003 and 2006); and preparing laboratory staff for an expected increase in specimens.

In recent years, the number of telephone triage systems has increased internationally in reaction to demands for rapid assessment and reassurance of people with health problems, and in an attempt to reduce the use of face-to-face health services, especially out of hours. Therefore, our results may be useful in other countries where there is a perceived need for real-time monitoring of common syndromes (both infectious and non-infectious). The ability of the telephone triage reporting software to map call data in real-time (not the case with NHS Direct) would also prove useful for analysing local demand for telephone triage, and for evaluating the impact of promotional activities.

### Strengths and weaknesses of the current spatial analysis

There are several weaknesses to this work. NHS Direct vomiting calls are likely to be caused by a range of viral and bacterial disease pathogens. This may partially hide, within the data, the true seasonality of norovirus reported to NHS Direct. It is possible that only new norovirus variants will have a substantial impact on the national burden of vomiting calls for this age group (≥ 5 years) displaying a pattern of national diffusion rather than regional raised incidence. For example, the novel norovirus genogroup II4 variant caused an increase and atypical summer peak in outbreaks across the UK and continental Europe [15] during 2002. Also, norovirus-infected individuals phoning NHS Direct may be reflective of norovirus outbreaks in community settings (for example, schools, food outlets), which exhibit little seasonal variation [6] and therefore do not exhibit clear seasonality within our telehealth data. Conversely, outbreaks in semi-closed institutional settings (for example, hospitals, residential homes) show a clear winter peak but these cases are unlikely to telephone NHS Direct because they are already receiving care. This work has not studied the spatiotemporal variation in vomiting calls about young children (in which there is the highest incidence of norovirus infection [5]). Calls about this group were excluded to remove the potentially confounding affect of rotavirus, which peaks during March in the UK. A study of the epidemic behaviour of rotavirus in the US demonstrated an annual South-West to North-East movement across the country [35]. Analysis of NHS Direct vomiting and diarrhoea calls about young children is required to explore further the spread of viral gastroenteritis in the UK, and respond to calls to supplement existing surveillance systems for infectious intestinal disease [14]. Unfortunately the norovirus outbreak data in our study does not represent outbreaks nationally and data from alternative laboratory or outbreak reporting systems will be sought for future work.

Common respiratory viruses, such as respiratory syncytial virus (RSV), may have contributed to the rise in fever calls (although RSV predominantly effects children younger than 5 years). Previous work estimating the contribution of a range of respiratory pathogens to NHS Direct calls found that RSV was responsible for approximately 15% of NHS Direct 'cough' calls [36]. Although a significant relationship was found between the seasonal variation in fever calls and influenza, no significant relationship was observed with RSV, parainfluenza or rhinovirus.

These analyses used only 151 relatively large spatial units (PCTs) to identify the approximate areas of rises in fever calls, rather than smaller units (postcode areas) to identify local disease transmission or hierarchical diffusion of disease from large to smaller urban centres. The lack of spatial precision means that these results are subject to the modifiable areal unit problem (MAUP) [37] whereby arbitrary units are used for spatial reporting. Our analysis suffers from what Armhein called the 'scale effect' of the MAUP [38]; the grouping of small areas into larger ones (postcode districts into PCTs). This had the effect of reducing the number of spatial units, increasing the average number of cases within each unit, but reducing the variation in incidence between units. We may have detected significant clustering at a sub-PCT level by using the smaller postcode district, although there were only 0.7 vomiting and 0.3 fever calls per week per postcode district during our study period. It is yet to be demonstrated that syndromic surveillance systems can *prospectively *and *consistently *provide early warning of localised outbreaks of disease. Their utility continues to be seen on a city-wide or regional basis at best. This study has used syndromic (proxy) measures of influenza and norovirus to identify a syndromic pattern consistent with contagious influenza diffusion at a PCT level, rather than onward transmission from an index case or single outbreak. PCTs are a relevant spatial unit for syndromic surveillance as primary care is likely to be among the first services to experience the effects of epidemic outbreaks.

The scan statistic employed here has previously been used for analyses of specific infections (for example, shigella [22] and *E. coli *[24]), ongoing public health surveillance (for example, New York City [2]), and monitoring disease during major sporting events (for example, the Kentucky Derby Festival [39]). Our work is novel in the use of the scan statistic for surveillance of telehealth calls, a data source that is infrequently used for syndromic surveillance purposes [40]. Although many NHS Direct calls reflect self-limiting disease (for example, influenza B, norovirus) this illness is still disruptive on a societal level and may go unreported by other healthcare surveillance systems. This work, for the first time, provides evidence that it is possible to describe the diffusion of national influenza outbreaks using telehealth data. When using the scan statistic, the underlying spatial variation in incidence is unimportant until it reaches a significant threshold level, clusters are detected, and an alert may be issued to public health teams. Other statistical approaches to deriving these critical values have been described [41,42]. An alternative approach, however, is kriging, where the entire range of disease incidence across a geographical area is modelled. Kriging uses interpolation techniques to estimate unknown point values of disease incidence from surrounding known point values, from which disease contours are generated [43]. The method has been employed successfully to describe the spread of the peak in influenza across Japan [44] and Europe [45]. For prospective surveillance, however, peak weeks are not known in advance and any global spatial clustering caused by differences in case definitions or reporting behaviour between regions would produce misleading results. The space-time scan statistic accounts for these spatial differences by using a control period from which to establish local baselines for each spatial unit. It may therefore be considered for future national surveillance initiatives, for example, the proposed hospital-based surveillance and 2012 Olympic planning in the UK.

Spatiotemporal analysis of NHS Direct syndromic data should supplement rather than replace the current analyses of regional call data [16] and other components of the UK influenza surveillance programme. It may help to identify sub-regional variation in influenza rates that may not manifest themselves within data from sentinel networks of GPs covering only a proportion of the population [46]. The added value demonstrated by our retrospective analyses was the early identification of a local rise in fever calls; a syndromic 'signal' that may require further investigation via conventional public health means. Used prospectively our analyses could be used to: warn hospitals, doctors, schools and nursing homes of impending problems; identify areas to introduce or enhance microbiological sampling; and describe geographic variation in influenza incidence throughout an epidemic period. We used fever calls regarding school-age children because we were studying a national influenza B outbreak. Spatial analyses of data about the NHS Direct 'colds and flu' syndrome are already used for national surveillance of ILI in adults [[16,47]]. Further winters' spatial analysis of respiratory syndromes is recommended to assess operational reliability of these results, and also to monitor influenza A strains associated with a high adult incidence.

## Conclusion

We have retrospectively described how NHS Direct syndromic call data can be used for geographical surveillance of influenza and norovirus infection in England. Spatiotemporal analyses of fever calls for the 5- to 14-year-old age group provided a timely and unique description of the evolution of a national influenza outbreak. These data provide the opportunity to present how a virus such as influenza spreads across the country affecting different localities to different degrees during the course of the influenza season. Such a tool therefore has utility for local services for both seasonal influenza and in the event of an influenza pandemic. In a similar way the tool may be useful for tracking norovirus, although the lack of reliable and consistent comparison data makes this more difficult to assess. In interpreting these results, care must be taken to consider other infectious and non-infectious causes of fever and vomiting.

To the best of the authors' knowledge this is the first published spatiotemporal analysis of national telehealth data used for surveillance purposes. The scan statistic should be considered analyses of telehealth data elsewhere. We will use these results to initiate prospective geographical surveillance of influenza in England.

## Competing interests

The authors declare that they have no competing interests.

## Authors' contributions

DLC – conducted literature search, developed research protocol, undertook all analyses, lead author on all drafts and final submission. GES – provided major scientific input to project aims and research protocol, 2^nd ^author. MR – provided specialist advice regarding influenza surveillance, suggestions and edits to discussion section. SL – provided specialist advice regarding telehealth systems, application of telehealth to surveillance, input into final two drafts. PG – provided scientific input regarding spatial epidemiology and statistical methods, and major edits of draft. All the authors have read and approved the final version of the manuscript.

## Pre-publication history

The pre-publication history for this paper can be accessed here:


